# Distinct signatures of the immune responses in low risk versus high risk neuroblastoma

**DOI:** 10.1186/1479-5876-9-170

**Published:** 2011-10-06

**Authors:** Madhu Gowda, Kamar Godder, Maciej Kmieciak, Andrea Worschech, Maria-Libera Ascierto, Ena Wang, Francesco M Marincola, Masoud H Manjili

**Affiliations:** 1 Department of Pediatrics, Children's Hospital of Richmond, Richmond, VA, USA; 2 Department of Microbiology and Immunology, Virginia Commonwealth University Massey Cancer Center, Richmond VA, USA; 3 Infectious Disease and Immunogenetics Section (IDIS), Department on Transfusion Medicine and Center for Human Immunology, National Institutes of Health, Bethesda, MD, USA; 4 Institute for Biochemistry, University of Wuerzburg, 97074 Wuerzburg, Germany

**Keywords:** Neuroblastoma, innate immunity, adaptive immunity, prognostic biomarkers

## Abstract

**Background:**

Over 90% of low risk (LR) neuroblastoma patients survive whereas less than 30% of high risk (HR) patients are long term survivors. Age (children younger than 18 months old) is associated with LR disease. Considering that adaptive immune system is well developed in older children, and that T cells were shown to be involved in tumor escape and progression of cancers, we sought to determine whether HR patients may tend to show a signature of adaptive immune responses compared to LR patients who tend to have diminished T-cell responses but an intact innate immune response.

**Methods:**

We performed microarray analysis of RNA extracted from the tumor specimens of HR and LR patients. Flow cytometry was performed to determine the cellular constituents in the blood while multiplex cytokine array was used to detect the cytokine profile in patients' sera. A HR tumor cell line, SK-N-SH, was also used for detecting the response to IL-1β, a cytokines which is involved in the innate immune responses.

**Results:**

Distinct patterns of gene expression were detected in HR and LR patients indicating an active T-cell response and a diminished adaptive immune response, respectively. A diminished adaptive immune response in LR patients was evident by higher levels of IL-10 in the sera. In addition, HR patients had lower levels of circulating myeloid derived suppressor cells (MDSC) compared with a control LR patient. LR patients showed slightly higher levels of cytokines of the innate immune responses. Treatment of the HR tumor line with IL-1β induced expression of cytokines of the innate immune responses.

**Conclusions:**

This data suggests that adaptive immune responses may play an important role in the progression of HR disease whereas innate immune responses may be active in LR patients.

## Background

Neuroblastoma, a tumor of the sympathetic nervous system is the most common cancer of infancy. The Children's Oncology Group stratifies patients into low risk (LR), intermediate risk (IR) or high risk (HR) categories based on age at diagnosis, International Neuroblastoma Staging System, tumor histopathology, DNA index and N-myc oncogene amplification status. Multiple biomarkers have been implicated in the prognosis of neuroblastoma, including N-myc amplification, DNA ploidy, Ferritin levels, neuron specific enolase, loss of chromosomes 1p, 11q or gain of 17q as well as TrkA and MDR associated proteins. Although N-myc is central to risk stratification, many metastatic neuroblastomas do not show amplification of this gene. In the absence of N-myc amplification, loss of heterozygosity of chromosome 11q was associated with a poor prognosis [1]. Today, there is no clear marker that can be used uniformly for all disease stages. There is also a consensus that the use of genetic data derived from diagnostic neuroblastoma tumors will remain central to patient treatment planning.

Age was shown to be an important prognostic factor such that patients older than 18 months were noted to have a worse prognosis than those who were younger [2-4]. The observation that children under 18 months of age do better than older children coincides with the development of the immune system. At a younger age the immune system depends primarily on the innate immunity whereas in older children the adaptive system has been well developed. In fact, several groups reported that cytokines/chemokines such as IL-1β, CXCL12, CXCR4 and IFN-γ which are involved in the innate immune responses play a critical role in neuronal differentiation associated with low-risk manifestation of the disease [5-9]. In vitro studies also underscored the innate immune responses by showing that human neuroblastoma cell lines were more susceptible to lysis by NK cells (innate immunity) than by the CD8+ T cells (adaptive immunity) [10]. Moreover, retinoic acid, currently being used in the treatment of minimal residual disease in HR neuroblastoma, was shown to promote innate immune responses and to some extent Th-1 responses leading to the inhibition of neuroblastoma [11,12]. However, it remains elusive whether Th-1 cells may be suppressed by an increased myeloid-derived suppressor cells (MDSC) or Tregs in LR patients. These findings support our hypothesis that a predominant innate immune response may be associated with LR neuroblastoma and a favorable outcome.

## Materials and methods

### Patient Samples

The study was approved by the Virginia Commonwealth University (VCU) Institutional Review Board (IRB) for collection of tumor and blood samples from patients being treated at VCU/Children's Hospital of Richmond (CHoR) for neuroblastoma. After obtaining an informed consent, tumor and blood samples were obtained at the time of diagnosis. Tumor biopsies and paired sera were obtained from the Children's Oncology Group tissue bank, Philadelphia. In order to identify distinct clinical risk phenotypes patients with intermediate risk neuroblastoma were excluded from the study. Patient characteristics are shown in Table 1.

**Table 1:** Patients' characteristics

Patients	Risk group	N-myc amplification	Ploidy	Stage	Shimada	Agae (m)	Sex
2	HR	Unknown	Unknown	4	UF	59	M

3	HR	No	> 1	4	UF	54	M

4	HR	Yes	> 1	4	F	22	F

15	HR	Yes	> 1	3	UF	114	F

10	HR	Yes	1	4	UF	24	M

13	HR	No	> 1	3	UF	18	F

6	HR	Yes	1	3	UF	55	M

17	HR	Yes	> 1	3	UF	23	M

18	HR	Yes	1	4	UF	20	M

1	LR	No	> 1	1	F	22	F

7	LR	No	> 1	2b	Unknown	19	M

11	LR	No	> 1	4s	F	5	M

12	LR	No	> 1	2b	F	4	F

9	LR	No	> 1	2a	F	3	M

14	LR	No	> 1	1	Unknown	6	M

5	LR	Unknown	Unknown	Unknown	Unknown	28	F

16	LR	No	Unknown	1	F	1	M

20	LR	No	1	2a	F	41	M

HR: high risk; LR: low risk; UF: unfavorable; F: favorable; M: male; F: female

### Tumor cell lines

The neuroblastoma cell line SK-N-SH isolated from a 4-year old HR neuroblastoma patient was obtained from ATCC. The cell line was cultured with ATCC formulated Eagle's Minimum Essential Media supplemented with 10% heat-inactivated fetal bovine serum.

### In vivo cell line studies

SK-N-SH cells (0.25 × 10^6 ^cells/well) were pulsed with IL-1β (200 ng/ml) in a total volume of 3 ml. Supernatant was collected after 24 or 72 hs and subjected to multiplex cytokine array analyses. The cells were detached and subjected to real-time PCR analysis of mRNA or flow cytometry analysis.

### Nucleic acid isolation and preparation

Total RNA (tRNA) from frozen tumor specimen was extracted after homogenization using Trizol reagent, according to the manufacturer's instructions (Invitrogen, Carlsbad, CA). The quality and quantity of RNA was assessed by Agilent Bioanalyzer 2000 (Agilent Technologies, Palo Alto, CA). For expression studies, tRNA was amplified into antisense RNA (aRNA) as previously described [13-15]. Universal reference RNA was derived from 6 normal donors' PBMC.

### Transcriptional analysis

Reference and test aRNA were directly labeled using ULS aRNA Fluorescent Labeling kit (Kreatech Diagnostics, Amsterdam, The Netherlands) with Cy3 for reference and Cy5 for test samples and co-hybridized to the 36 k human oligo array slides. After 20 hs incubation at 42°C the arrays were washed, dried and scanning using the Agilent scanner.

### Data processing and statistical analysis

Transcriptional data were uploaded to the mAdb databank http://nciarray.nci.nih.gov and further analyzed using BRBArrayTools developed by the Biometric Research Branch, NCI http://linus.nci.nih.gov/BRB-ArrayTools.html[16], Partek Genomics Suite (St Louis, MO) or TreeView software [17]. The complete dataset, was filtered (50% gene presence across all experiments) to enrich for informative transcripts obtaining a total of 27,330 transcripts. Gene ratios were average corrected across experimental samples and displayed according to uncentered correlation algorithm. Unsupervised analysis was performed for class confirmation using the BRB ArrayTools and Stanford Cluster Program.

Student's t test (cut off p_2 _value ≤ 0.01) applied to the filtered data set was used to compare the LR-patients with HR-patients. The analysis identified 408 genes differentially expressed between the two groups (global permutation p value = 0.01). Among them, 91 were up regulated and 317 down regulated in patients with High Risk neuroblastoma. Functional gene network analysis was performed using the Ingenuity Pathway Analysis system (IPA) which transforms large data sets into a group of relevant networks containing direct and indirect relationships between genes based on known interactions in the literature. Gene function interpretation was based on Ingenuity Pathway Analysis (IPA, Ingenuity Systems).

### Flow Cytometry

A three-color staining and FACS analyses were performed as previously described by our group [18]. Extracellular staining was performed using anti-human Abs from Biolegend: FITC-labeled anti-HLA-DR, CD62, CD56 and CD25; PE-labeled anti-CD11b, CD44, NKG2D and CXCR4, and PE/CY5-labeled anti-CD4, CD8 and CD3. Appropriate isotype control Abs were used to exclude nonspecific binding. Foxp3 intracellular staining was done using a PE anti-human Foxp3 Flow Kit (Biolegend, clone 206D) according to the manufacturer's protocol. Apoptosis was determined by staining of cells with Annexin V and PI (BD Pharmingen).

### Reverse transcriptase and real-time PCR

The cDNA was prepared from 2 μg of total RNA using the Super script II Kit (Invitrogen) with a T17 oligonucleotide primer. cDNA synthesis was completed at 42°C for 2h. Sybr green-based SensiMix (Bioline, Taunton, MA) was used according to manufacturer's instructions, and real time PCR was performed using the Bio-Rad's real time PCR detection system.

### Multiplex cytokine array

Sera from the blood were used to detect a panel of 12 cytokines (IL-1β, IL-2, IL-4, IL-6, IL-10, IL-17α, IFN-γ, TNF-α, TGF-β, MCP-1, GM-CSF and RANTES) using the Bio-Plex Human Multiplex Cytokine Assay from Bio-Rad as per the protocol from the company. Cytokine array of supernatants of cell studies were sent to Ocean Ridge Biosciences LLC (Palm Beach Gardens, FL) for analysis.

## Results

### Differential gene expression profiles at the tumor site are associated with HR vs. LR neuroblastoma

**Figure 1:**
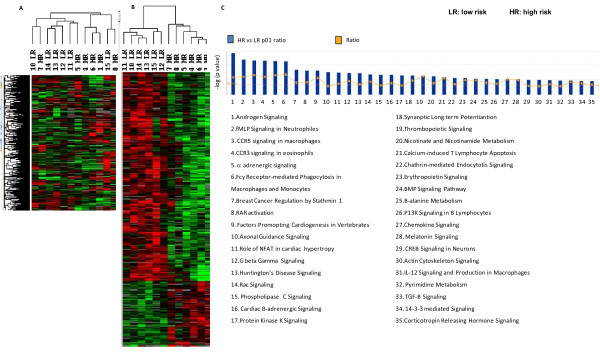
**Differential gene expression profile in tumor lesions of patients with HR vs. LR neuroblastoma**. A) Unsupervised cluster analysis of LR vs. HR patients. B) Supervised cluster analysis (Student *t *test, p < 0.01 and fold change > 3) comparing LR vs. HR. C) Canonical pathway analysis shows the significant genes and pathways that differ between HR and LR neuroblastoma.

We have previously reported that differential patterns of gene expression at the tumor site, which include tumor cells and infiltrating immune cells, were associated with the immune-mediated rejection or recurrence of mouse mammary carcinoma [19] as well as human breast carcinoma [20]. Therefore, we sought to determine whether a similar approach, i.e. differential patterns of the immune function genes at the tumor site, may be associated with LR vs. HR neuroblastoma. Unsupervised cluster analysis segregated LR and HR patients with an exception for patients # 7 and 15 (Figure 1A). An unpaired Student *t *test with a cut-off set at p < 0.01 identified 408 genes differentially expressed between LR and HR tumors (permutation p value = 0.01) of which 91 and 317 genes were up-regulated and down-regulated in HR vs. LR, respectively (Figure 1B). Canonical pathway analysis revealed that CCR3, CCR5 and IL-12 signaling pathways as well as Fcγ receptor-mediated antigen uptake were upregulated in the HR patients (Figure 1C).

### LR patients tend to have skewed innate immune responses compared to HR patients

**Figures 2:**
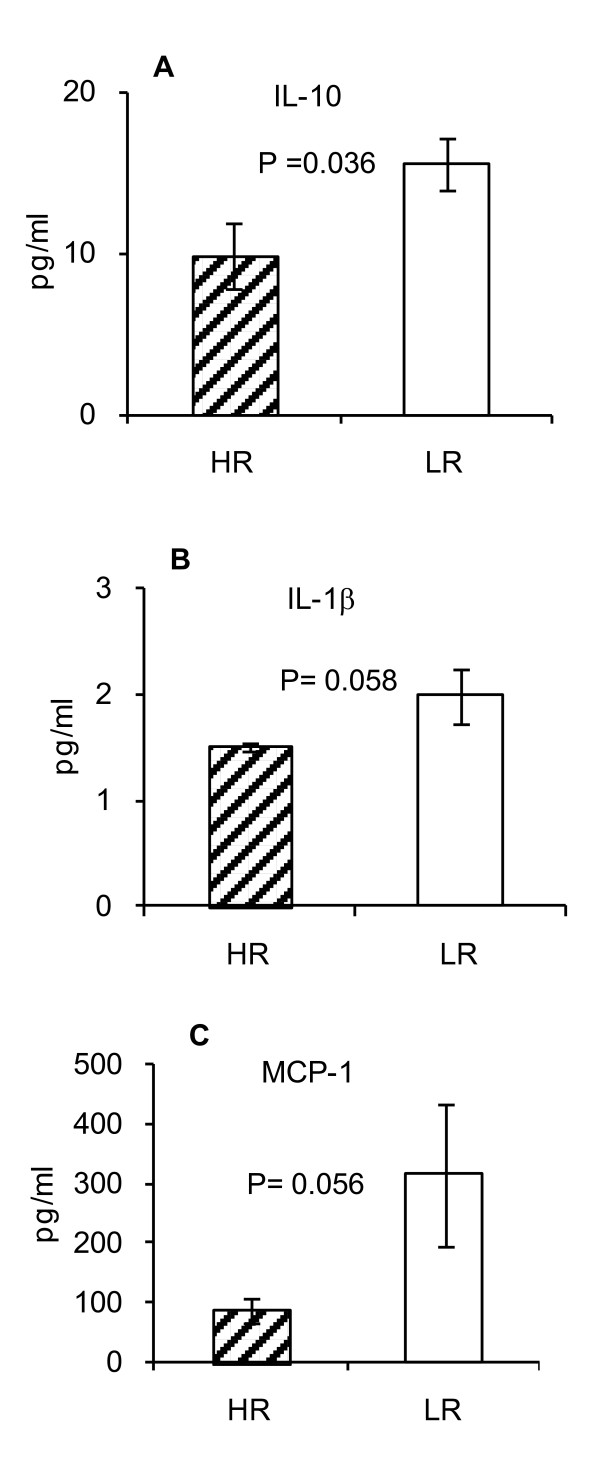
**LR patients show higher titers of IL-10, IL-1β and MCP-1 in their sera**. Multiplex cytokine array analyses of the sera of patients with LR and HR neuroblastoma for detected higher levels of IL-10 (A) IL-1β (B), and MCP-1 (C) in LR patients. Data represent 4-6 HR and 6-7 LR patients.

In order to determine whether patients with LR neuroblastoma may exhibit an active innate but not an adaptive immune response compared to HR patients, we performed multiplex cytokine array analysis of the sera. We used a kit which detects the following cytokines (IL-1β, IL-2, IL-4, IL-6, IL-10, IL-17α, IFN-γ, TNF-α, TGF-β, MCP-1, GM-CSF and RANTES). As shown in Figure 2A-B, IL-10 which is involved in counteracting CD8+ T cell and CD4+ Th-1 cell responses was significantly higher in LR vs. HR patients (p = 0.036). In addition, cytokines involved in the innate immune responses such IL-1β and MCP-1 were appreciably higher in LR vs. HR patients (Figure 2B-C; p = 0.056 and 0.058, respectively).

### HR patients tend to have skewed adaptive immune responses compared to LR patients

**Figures 3:**
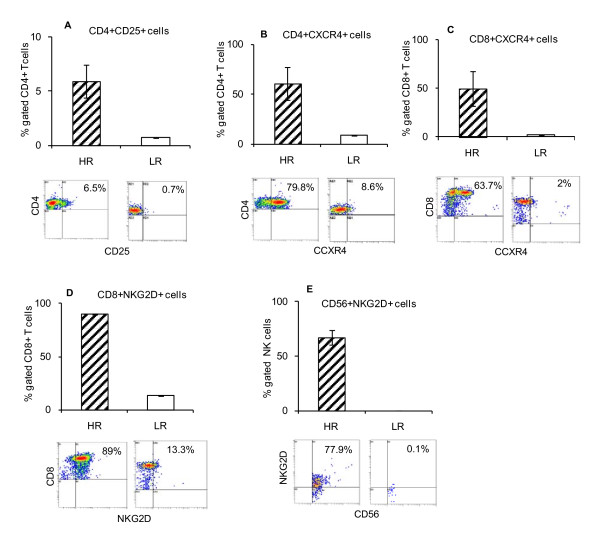
**HR patients show an increased adaptive immunity compared to LR patients**. Flow cytometric analysis of PBMC of patients with HR (n = 3) and LR (n = 1) neuroblastoma.

Flow cytometry analyses of PBMC showed that HR patients had higher frequency of CD4+CD25+ T cells, CD4+CXCR4+ T cells, CD8+CXCR4+ T cells, and CD8+NKG2D+ compared to LR patients (Figure 3A-D). In addition, CD56+NKG2D+ NK cells also showed higher frequency in HR compared to LR patients (Figure 3E). All these cells, except for NK cells, are involved in the adaptive immune responses.

Induction of pro-inflammatory cytokines during the innate immune responses could facilitate the induction of adaptive immune responses such that increased levels of IL-10 in LR patients may not be enough to suppress an adaptive immune activation in these patients. Therefore, we sought to determine whether adaptive immune system might be under additional suppression mechanisms in patients with LR but not HR neuroblastoma. We looked at the presence of CD4+CD25+FoxP3+ Tregs and MDSC (CD33+CD11b+HLA-DR-) in the peripheral blood of patients. Frequency of Tregs was negligible in both LR and HR patients (data not shown). However, all the HR patients showed lower levels of MDSC compared to a control LR patient (Additional file 1). Since neuroblastoma is a very rare disease, we were not able to accrue more than one patient with LR disease during this study.

### IL-1β induces MCP-1 production as well as GM-CSF and TNF-α

**Figure 4:**
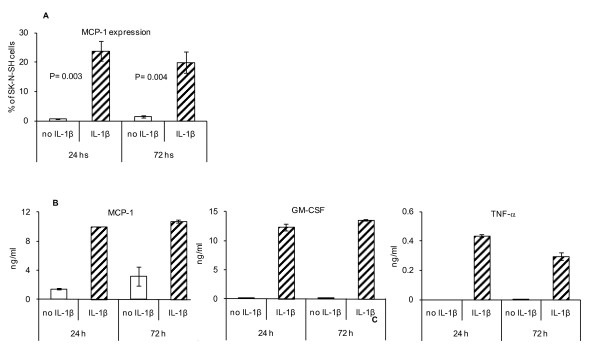
**IL-1β induces expression of pro-inflammatory cytokines in SK-N-SH tumor cell line**. A) Flow cytometry analysis of SK-N-SH tumor cell line in the absence or presence of IL-1β after 24 hs and 72 hs culture in vitro. Data represent four independent experiments. B) Multiplex cytokine array analysis of supernatants of SK-N-SH tumor cells cultured in the absence or presence of IL-1β after 24 hs and 72 hs for the detection of MCP-1, GM-CSF and TNF-α.

To determine whether the pro-inflammatory cytokine IL-1β may induce the expression of cytokines of the innate immune response in a HR tumor cell line, SK-N-SH cells were cultured in the presence or absence of IL-1β (600 ng/0.25 × 10^6 ^cells) for 24 or 72 hs, and supernatants were subjected to multiplex cytokine array analysis while cells were analyzed by flow cytometry. As shown in Figure 4A, IL-1β induced the expression of MCP-1 in the cells as well as an increased expression of MCP-1, GM-CSF and TNF-α in the supernatant (Figure 4B) with no effect on the expression of IL-10 (data not shown).

## Discussion

We investigated the status of the adaptive and innate immune responses in patients with HR or LR neuroblastoma. Patients with HR showed increases in the number of cells involved in adaptive immune responses as well as lower levels of IL-10 and MDSC, compared with LR patients. In addition, cytokines involved in the innate immune responses including IL-1β and MCP-1 were increased in LR patients. Treatment of a HR tumor cell line with IL-1β induced the expression of pro-inflammatory cytokines involved in the innate immune responses, including MCP-1, GM-CSF and TNF-α. Our data suggest a favorable prognostic value of the signatures of immune function genes associated with the innate immune response, such as increased IL-1β and MCP-1 as well as a diminished adaptive immune response, evidenced by increased levels of MDSC, IL-10 and decreased expression of NKG2D and CXCR4.

IL-1β and MCP-1 are involved in pro-inflammatory responses, and are components of non specific innate immune responses. Increased expression of these molecules in LR patients underscores their importance in a favorable prognosis. Since IL-1β has been shown to induce the expression of MCP-1 [21] leading to chronic inflammation, we hypothesized that IL-1β-may induce the expression of MCP-1 and IL-10 in HR tumor cells. We showed an increased expression of MCP-1, GM-CSF and TNF-α in the HR tumor cell line pulsed with IL-1β, both by flow cytometry and cytokine array analyses. GM-CSF has been shown to promote the development of MDSC and in turn suppress adaptive immune responses [22]. A higher frequency of CD8+CXCR4+ T cells in the peripheral blood of HR patients may facilitate infiltration of T cells into the tumor site [23]. However, such an increased infiltration of T cells failed to protect HR patients. This may be because of a dual function of CD8+ T cells, i.e. inducing epigenetic changes in the tumors leading to tumor escape and a worse prognosis [24] as well as a direct cytotoxic effect on tumor cells. For instance, we have shown that CD8+ T cells can induce epithelial to mesenchymal transition (EMT) as well as HER-2/neu antigen loss, leading to tumor escape in breast cancer model [25,26]. Others also reported that T cells can induce tumor escape in a variety of tumor models including CT-26 colon carcinoma [27], renal cell carcinoma [28], Uveal melanoma [29] and breast cancer patients [30].

NKG2D is an activating receptor expressed on activated CD8+ T cells and NK cells. Signalling by NKG2D has been shown to be involved in the activation of T cells against the tumors [31]. Of note was the relative absence of activated NK cells (CD65+NKG2D+) in the circulation of LR patients. NK cells play a key role in innate immunity, and it was surprising to note higher levels of CD56+NKG2D+ cells in HR patients. Higher expression of MCP-1 in LR tumors could induce infiltration of NK cells to the tumor site and as a result reduce circulating NK cells [32].

Regrouping the microarray data based on the percentage of infiltrating immune cells would further refine the differential expression of the immune function genes in HR vs. LR patients. However a limitation in our study was that because of the rare nature of the disease and limited access to sufficient number of patients, many of our samples were received from outside the institution. These samples contained RNA so that the percent of infiltrating cells could not be determined and regrouping for microarray analysis was not possible. However our data still show very clear segregation between the two groups.

It has been reported that pro-inflammatory products can cause impairment of DNA synthesis in neuroblastoma cell lines [33] leading to cell death.

While a single gene or cytokine may not be able to independently be a prognosticator; the combination of differentially expressed immune function genes and the pattern of cellular and cytokine responses can be used to generate a 'signature' pattern for HR vs. LR neuroblastoma. A signature of the innate immune responses is important in lieu of reports showing that pro-inflammatory products can cause impairment of DNA synthesis in neuroblastoma cell lines [33] leading to cell death. MDSC stand out as a distinct difference between the two groups and it can be added to the current prognostication parameters, though it needs to be further validated by using a large number of samples because we were not able to include more than one LR patient for the detection of MDSC in the circulation due to the rate nature of LR disease. One of our limitations was a small sample size due to the nature of the diseases and the relatively higher frequency of HR disease.

## Conclusions

HR patients tend to have active T cell responses whereas LR patients showed reduced T cell responses and higher levels of cytokines involved in the innate immune responses. It remains to be determined whether treatment of HR tumors with pro-inflammatory cytokines such as IL-1β may convert HR tumors into LR tumors.

## Competing interests

The authors declare that they have no competing interests.

## Authors' contributions

**MG **carried out molecular studies, in vitro cell culture studies and flow cytometry, drafted the manuscript, analyzed the data, participated in the design and coordination of the study. KG participated in the design and coordination of the study, drafted the manuscript. MK carried out molecular studies, in vitro cell culture studies and flow cytometry, drafted the manuscript, analyzed the data. AW carried out microarray analysis, participated in data analysis and drafting the manuscript. MLA carried out microarray analysis, participated in data analysis and drafting the manuscript. EW participated in data analysis and drafting the manuscript, performed statistical analysis, participated in the design and coordination of the study. FMM participated in data analysis and drafting the manuscript, performed statistical analysis, participated in the design and coordination of the study. MHM designed the experiments, drafted the manuscript, analyzed the data, participated in the coordination of the study, conceived the study. All authors read and approved the final manuscript.

## Supplementary Material

Additional file 1 **LR patients show an increased MDSC in their circulation compared to HR patients**. Flow cytometric analysis of PBMC of patients with HR (n = 4) and LR (n = 1) neuroblastoma. Click here for file

## References

[B1] London WB Castleberry RP Matthay KK Look AT Seeger RC Shimada H Thorner P Brodeur G Maris JM Reynolds CP Cohn SL Evidence for an age cutoff greater than 365 days for neuroblastoma Risk group stratification in the children's oncology group J Clin Oncol 2005 23 27 6495 6465 10.1200/JCO.2005.05.571 16116153

[B2] Breslow N McCann B Statistical estimation of prognosis for children with neuroblastoma Cancer Res 1971 31 12 2098 2013 5120301

[B3] Evans AE Staging and treatment of neuroblastoma Cancer 1980 45 suppl 7 1799 1802 29603175 10.1002/cncr.1980.45.s7.1799

[B4] Weinstein LJ Katzenstein MH Cohn LS Advances in diagnosis and treatment of neuroblastoma The Oncologist 2003 8 278 292 10.1634/theoncologist.8-3-278 12773750

[B5] Wang X Fu S Wang Y Yu P Hu J Gu W Xu XM Lu P Interleukin-1beta mediates proliferation and differentiation of multipotent neural precursor cells through the activation of SAPK/JNK pathway Mol Cell Neurosci 2007 36 3 343 54 10.1016/j.mcn.2007.07.005 17822921

[B6] Ideguchi M Shinoyama M Gomi M Hayashi H Hashimoto N Takahashi J Immune or inflammatory response by the host brain suppresses neuronal differentiation of transplanted ES cell-derived neural precursor cells J Neurosci Res 2008 86 9 1936 1943 10.1002/jnr.21652 18335525

[B7] Kampmann E Johann S van Neerven S Beyer C Mey J Anti-inflammatory effect of retinoic acid on prostaglandin synthesis in cultured cortical astrocytes J Neurochem 2008 106 1 320 332 10.1111/j.1471-4159.2008.05395.x 18394023

[B8] Peng H Kolb R Kennedy JE Zheng J Differential expression of CXCL12 and CXCR4 during human fetal neural progenitor cell differentiation J Neuroimmune Pharmacol 2007 2 3 251 258 10.1007/s11481-007-9081-3 18040858 PMC2169289

[B9] Kim SJ Son TG Kim K Park HR Mattson MP Lee J Interferon-gamma promotes differentiation of neural progenitor cells via the JNK pathway Neurochem Res 2007 32 8 1399 1406 10.1007/s11064-007-9323-z 17415631

[B10] Main EK Lampson LA Hart MK Kornbluth J Wilson DB Human neuroblastoma cell lines are susceptible to lysis by natural killer cells but not by cytotoxic T lymphocytes J Immunol 1985 135 1 242 246 3158702

[B11] Cetinkaya C Hultquist A Su Y Wu S Bahram F Pahlman S Guzhova I Larsson LG Combined IFN-gamma and retinoic acid treatment targets the N-Myc/Max/Mad1 network resulting in repression of N-Myc target genes in MYCN-amplified neuroblastoma cells Mol Cancer Ther 2007 6 10 2634 2641 10.1158/1535-7163.MCT-06-0492 17938259

[B12] Austenaa LM Ross AC Potentiation of interferon-gamma-stimulated nitric oxide production by retinoic acid in RAW 264.7 cells J Leukoc Biol 2001 70 1 121 129 11435494

[B13] Krausa P Browning MJ A comprehensive PCR-SSP typing system for identification of HLA-A locus alleles Tissue Antigens 1996 47 3 237 244 10.1111/j.1399-0039.1996.tb02547.x 8740775

[B14] Wang E Miller LD Ohnmacht GA Liu ET Marincola FM High-Fidelity mRNA amplification for gene profiling Nat Biotechnol 2000 18 4 457 459 10.1038/74546 10748532

[B15] Wang E RNA amplification for successful gene profiling analysis J Transl Med 2005 3 28 10.1186/1479-5876-3-28 16042807 PMC1201175

[B16] Jin P Zhao Y Ngalame Y Panelli MC Nagorsen D Monsurró V Smith K Hu N Su H Taylor PR Marincola FM Wang E Selection and validation of endogenous reference genes using a high throughput approach BMC Genomics 2004 5 1 55 10.1186/1471-2164-5-55 15310404 PMC516027

[B17] Simon R Lam A Li MC Ngan M Menenzes S Zhao Y Analysis of Gene Expression Data Using BRB-Array Tools Cancer Inform 2007 3 11 17 19455231 PMC2675854

[B18] Kmieciak M Gowda M Graham L Godder K Bear HD Marincola FM Manjili MH Human T cells express CD25 and Foxp3 upon activation and exhibit effector/memory phenotypes without any regulatory/suppressor function J Transl Med 2009 7 89 10.1186/1479-5876-7-89 19849846 PMC2770477

[B19] Worschech A Kmieciak M Knutson KL Bear HD Szalay AA Wang E Marincola FM Manjili MH Signatures associated with rejection or recurrence in HER-2/neu-positive mammary tumors Cancer Res 2008 68 7 2436 2446 10.1158/0008-5472.CAN-07-6822 18381452 PMC2478745

[B20] Ascierto ML Kmieciak M Idowu MO Manjili R Zhao Y Grimes M Dumur C Wang E Ramakrishnan V Wang XY Bear HD Marincola FM Manjili MH A signature of immune function genes associated with recurrence-free survival in breast cancer patients Breast Cancer Res Treat 2011 in press 10.1007/s10549-011-1470-x PMC3431022 21479927

[B21] Yoshimura H Nakahama K Safronova O Tanaka N Muneta T Morita I Transforming growth factor-beta stimulates IL-1beta-induced monocyte chemo attractant protein-1 expression in human synovial cells via the ERK/AP-1 pathway Inflamm Res 2006 55 12 543 549 10.1007/s00011-006-5144-9 17039283

[B22] Morales JK Kmieciak M Knutson KL Bear HD Manjili MH GM-CSF is one of the main breast tumor-derived soluble factors involved in the differentiation of CD11b-Gr1- bone marrow progenitor cells into myeloid-derived suppressor cells Breast Cancer Res Treat 2010 123 1 39 49 10.1007/s10549-009-0622-8 19898981 PMC3095485

[B23] Gouwy M Struyf S Berghmans N Vanormelingen C Schols D Van Damme J CXCR4 and CCR5 ligands cooperate in monocyte and lymphocyte migration and in inhibition of dual-tropic (R5/X4) HIV-1 infection Eur J Immunol 2011 41 4 963 973 10.1002/eji.201041178 21381021

[B24] Dunn GP Old LJ Schreiber RD The three Es of cancer immunoediting Annu Rev Immunol 2004 22 329 360 10.1146/annurev.immunol.22.012703.104803 15032581

[B25] Kmieciak M Knutson KL Dumur CI Manjili MH HER-2/neu antigen loss and relapse of mammary carcinoma are actively induced by T cell-mediated anti-tumor immune responses Eur J Immunol 2007 37 3 675 685 10.1002/eji.200636639 17304628 PMC3732067

[B26] Santisteban M Reiman JM Asiedu MK Behrens MD Nassar A Kalli KR Haluska P Ingle JN Hartmann LC Manjili MH Radisky DC Ferrone S Knutson KL Immune-induced epithelial to mesenchymal transition in vivo generates breast cancer stem cells Cancer Res 2009 69 7 2887 2895 10.1158/0008-5472.CAN-08-3343 19276366 PMC2664865

[B27] Beatty GL Paterson Y IFN-gamma can promote tumor evasion of the immune system in vivo by down-regulating cellular levels of an endogenous tumor antigen J Immunol 2000 165 10 5502 5508 11067903 10.4049/jimmunol.165.10.5502

[B28] Hall VL Subleski J BacK TC Gruys ME Shorts-Cary L Weiss JM Wiltrout RH Friend or Foe? IFN Mediates Pro-Metastatic Gene Expression in the Tumor Microenvironment J Immunol 2007 178 48.34

[B29] Hallermalm K Seki K De Geer A Motyka B Bleackley RC Jager MJ Froelich CJ Kiessling R Levitsky V Levitskaya J Modulation of the tumor cell phenotype by IFN-gamma results in resistance of uveal melanoma cells to granule-mediated lysis by cytotoxic lymphocytes J Immunol 2008 180 6 3766 3774 18322182 10.4049/jimmunol.180.6.3766

[B30] Matkowski R Gisterek I Halon A Lacko A Szewczyk K Staszek U Pudelko M Szynglarewicz B Szelachowska J Zolnierek A Kornafel J The prognostic role of tumor-infiltrating CD4 and CD8 T lymphocytes in breast cancer Anticancer Res 2009 29 7 2445 2451 19596912

[B31] Maccalli C Scaramuzza S Parmiani G TNK cells (NKG2D+ CD8+ or CD4+ T lymphocytes) in the control of human tumors Cancer Immunol Immunother 2009 58 5 801 808 10.1007/s00262-008-0635-x 19089424 PMC11030286

[B32] Metelitsa LS Wu HW Wang H Yang Y Warsi Z Asgharzadeh S Groshen S Wilson SB Seeger RC Natural killer T cells infiltrate neuroblastomas expressing the chemokine CCL2 J Exp Med 2004 199 9 1213 1221 10.1084/jem.20031462 15123743 PMC2211904

[B33] Werner EJ Walenga RW Dubowy RL Boone S Stuart MJ Inhibition of human malignant neuroblastoma cell DNA synthesis by lipoxygenase metabolites of arachidonic acid Cancer Res 1985 45 2 561 563 3917850

