# Supplementary figures and images for: Distinct signatures of the immune responses in low risk versus high risk neuroblastoma
Source: J Transl Med. 2011 Oct 6;9:170. doi: 10.1186/1479-5876-9-170 (PMC3195752; doi:10.1186/1479-5876-9-170)

## Slide 1
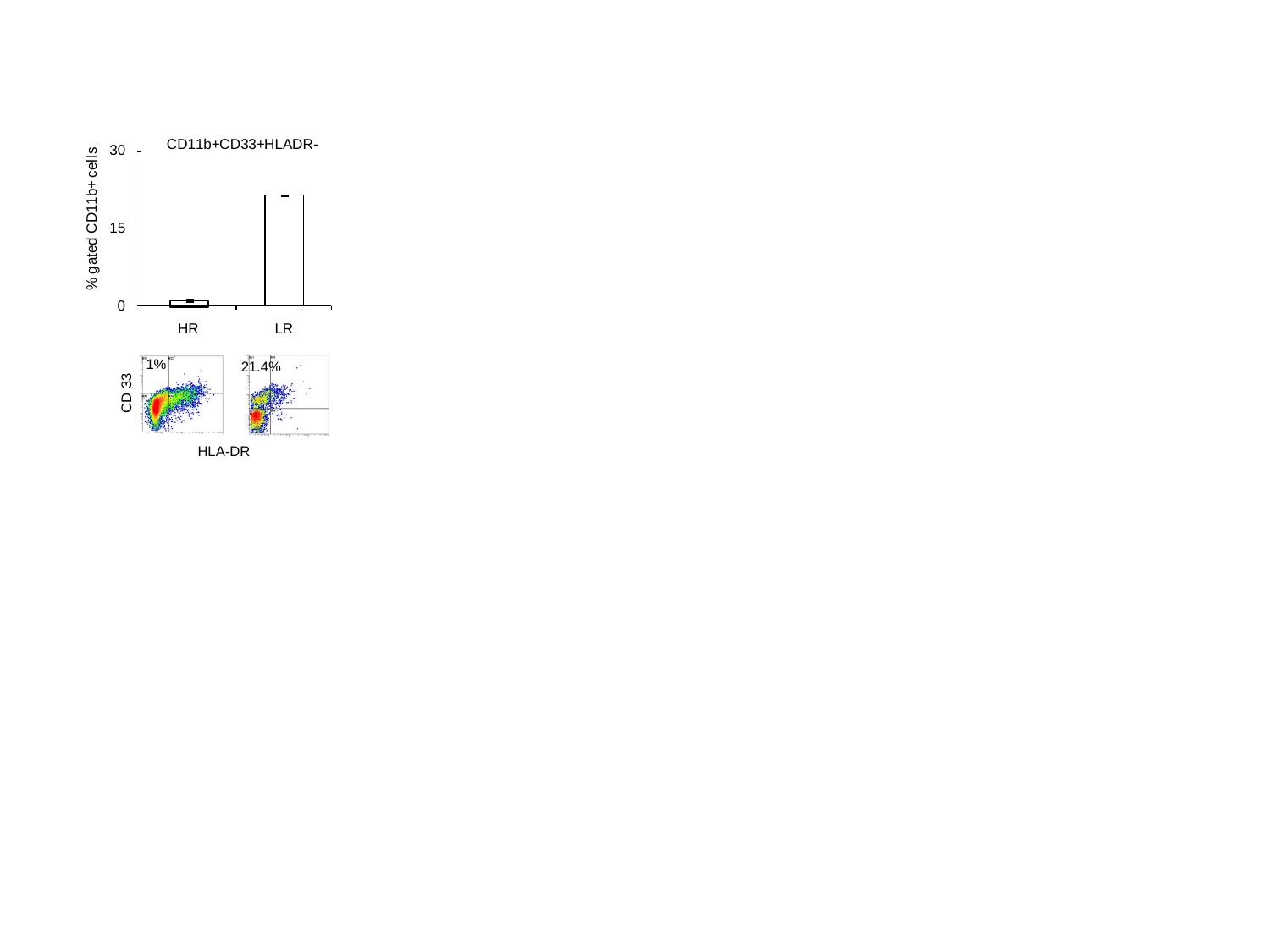

1%
21.4%
CD 33
HLA-DR

Supplement: Additional file 1 — LR patients show an increased MDSC in their circulation compared to HR patients. Flow cytometric analysis of PBMC of patients with HR (n = 4) and LR (n = 1) neuroblastoma. [file 1479-5876-9-170-S1.PPT]
